# Concurrence of Multiple Sclerosis and Brain Tumors

**DOI:** 10.3389/fneur.2015.00040

**Published:** 2015-03-04

**Authors:** Domenico Plantone, Rosaria Renna, Emilia Sbardella, Tatiana Koudriavtseva

**Affiliations:** ^1^Unit of Neurology, Multiple Sclerosis Center, Regina Elena National Cancer Institute, IFO, Rome, Italy

**Keywords:** multiple sclerosis, brain tumors, glioblastoma, oligodendroglioma, astrocytoma

## Introduction

Multiple sclerosis (MS) is a chronic demyelinating inflammatory disease of the central nervous system (CNS) with multi-factorial pathogenesis that includes genetic and environmental factors. A primary brain tumor is a neoplasm developing from the cells of the brain. There is high heterogeneity of primary brain tumors (about 100 different types); however, most of them develop from the glial cells.

Although several types of brain tumors have been widely described in association with MS (1–20), including astrocytoma (5, 9), oligodendroglioma (12, 17), and glioblastoma (3, 11, 14, 18), it is not clear whether their occurrence is accidental or consequent to causal events. Moreover, it is not completely defined if MS and brain tumors, when associated, have a different course. The true incidence of brain tumors in MS patients is difficult to define because the diagnosis of a brain tumor in MS patients may seem more frequent than in the general population due to frequent neuroimaging scans performed in these patients (21). At the same time, pseudo-tumoral MS lesions may resemble gliomas, and conversely, early stage gliomas may resemble MS. Brain tumors in MS patients may be diagnosed later or even post-mortem (22), especially in patients with progressive MS, since the new symptoms may be attributed to the gradual clinical progression of MS rather than to the slow growing of tumor itself (23). A recent study reported that MS patients have a decreased overall cancer risk, but an increased risk for brain tumor (24). If immunosuppressive treatment for MS might promote cancerogenesis is still matter of debate, it is difficult to explain on this basis why MS patients have a decreased overall cancer risk and an increased risk only for brain and genitourinary tract tumors (24). A successive systematic analysis showed no increased or decreased risks for glioma in MS patients, while an increased risk was found for meningioma, as a result of incidental findings (25). Moreover, several autoimmune diseases influence negatively the survival in glioma and meningioma, likely due to pre-existent disability or treatment limitations (25).

## Differential Diagnosis between MS and Brain Tumors

A first issue emerging in case of concurrence of MS and brain tumors is the differential diagnosis since MS plaques may resemble gliomas and vice-versa (26). Although brain tumors associated with MS have their usual distribution at frontal and temporal lobes (27), the localization criteria are not helpful for the differential diagnosis. Therefore, the appearance of uncommon neurological symptoms in MS patients should suggest the need for more extensive investigations in order to exclude overlapping pathologies (28). In addition to brain tumors, the differential diagnosis of pseudo-tumoral brain lesions includes infectious, neoplastic (in particular primary CNS lymphomas and metastatic cancers), congenital, metabolic or vascular diseases, and non-MS idiopathic inflammatory demyelinating diseases as well (for instance, neuromyelitis optica, opticospinal MS in Asian populations, acute disseminated encephalomyelitis) (29). They differ from MS in course, pathophysiology, treatment, and prognosis. In 2008, the Task Force on Differential Diagnosis in MS has defined major, intermediate, and minor red flags consisted in informative symptoms, signs, and assays indicative, respectively, of non-MS diagnosis, uncertainty or possibility that an MS diagnosis is not excluded (29). The major red flags, for example, for lymphoma, are represented by persistent Gd-enhancement, continued enlargement of lesions, simultaneous enhancement of all lesions, marked asymmetry of white matter lesions, headache, or meningismus.

Among the diagnostic tools that have been proposed to differentiate MS from tumors, there are also some blood indicators such as inflammatory transcription factors of the peripheral blood mononuclear cells (18). However, they are more useful in expressing patients’ immunological status than in making a proper differential diagnosis between MS and brain tumors. To date, the non-conventional MRI techniques such as spectroscopy, positron emission tomography, and CNS biopsy remain the most useful tools for the differential diagnosis.

## Possible Causal Relationship between Brain Tumors and MS

Whether the concurrence of MS and glioma may be explained by causal relationship or by coincidence is still matter of debate (17, 25). In 1973, it was observed that despite the rare concurrence of such relatively common conditions some pathological features, especially the frequent contiguous relationship between tumor and plaque, suggested a closer association between these pathologies (30). It was hypothesized that in some MS patients an unknown factor, hereditary or acquired, may stimulate the neoplastic transformation of reactive astrocytes. Moreover, it was supposed a causative role for a bipotential cytolytic-oncogenic agent such as Papova virus (30). Almost 20 years later, another study, based on the most extensive literature, came to similar conclusions (31). A transformation of a mega-plaque into an ependymoma added new evidence in favor to a cause–effect relationship between MS and brain tumors (32). It can be hypothesized that MS lesions may transform into a tumor likely due to circumscribed increased proliferation ratio induced by MS remyelinating processes since the neurotropic growth factors promoting the proliferation and survival of oligodendrocytes have the beneficial effect on the clinical, pathological, and molecular manifestations of autoimmune demyelination in experimental models (33, 34). It was also found a re-expression of a developmental gene in chronic lesions with the highest levels of their products correlating with remyelination (35). The recapitulation of ontogenetic events during myelin repair has been supposed as normal adult CNS and non-MS material showed very low levels of such gene products, while fetal human CNS tissue showed high levels. Therefore, the possibility of common rare underlying genetic factors in both tumors and MS is possible, but so far there are no sufficient data to definitely confirm it. Furthermore, owing to high heterogeneity of primary brain tumors, the research on their genetic alterations is not simple. Interestingly, the recently identified MS risk genes mainly belong to the immune system (36), and alterations in innate immunity-related genetic regions have been associated with an increased risk of adult glioma (37). It may be also hypothesized that aberrant epigenetic mechanisms, such as DNA methylation and histone protein modifications, could be involved in both MS and tumor pathogenesis (38). Methylated gene promoters are silenced, while unmethylated ones can be active reflecting, albeit imprecisely, gene expression. Certain regions of the brain in MS patients show abnormally methylated genes with a specific profile or decreased methylation, for example, of cytosine of the gene encoding the myelin enzyme peptidylarginine deiminase-2 promoter in MS normal-appearing white matter (39). Still more, DNA methylation is altered in tumors and may represent a predictive factor of response to specific drugs. In particular, the O6-methylguanine-DNA methyltransferase promoter hypermethylation was found to be strongly associated with partial or complete clinical response (40–42). However, no possible causal epigenetic mechanisms between brain tumors and MS could be inferred by these limited data.

Finally, it is possible to speculate that a common viral agent is involved in the pathogenesis of these diseases. After 30 years from the first supposition of a possible Papova virus role in both disorders (30), the post-mortem examination of an immunocompetent patient with MS plaques and a glioblastoma multiforme provided molecular evidence of the association of human polyomavirus JC virus (JCV) of Papova virus family with these concurrent pathologies (14). PCR analysis revealed the presence of viral DNA in demyelinated plaques and within the tumor, while immunohistochemistry showed the detection of the viral early protein, T-antigen, and the cellular tumor suppressor protein, p53, only in the nuclei of neoplastic cells. Conversely, the expression of T-antigen, but not of p53, was observed in astrocytes and neuronal cells of the cortex juxtaposed to the MS plaque. No productive replication of JCV was identified in both tumor and MS lesions since the examination of viral late gene expression by immunohistochemistry showed no evidence for viral capsid proteins. Furthermore, some authors detected the presence of JCV in samples derived from several types of neural and non-neural human tumors (43–50), and several studies highlighted its potential role in a broad range of animal models and human carcinogenesis (51–54).

JC virus was identified as the etiologic agent of progressive multifocal leukoencephalopathy, first diagnosed only in immunocompromised patients or those suffering from leukemia, but now representing a serious complication of Natalizumab treatment in MS (55, 56). JCV is very common in the general population infecting 70–90% of humans (57), and a higher rate of JCV seroconversion compared to than expected has been observed in MS patients treated with Natalizumab (58). JC viral genome has been detected in normal brain tissue (59), therefore, the latent JCV-DNA antigens expressed at low levels in the CNS have been proposed as possible targets of pathogenetic immune response in MS (60). Initially, the JCV-DNA was found neither in the urine (61) nor in the brain tissue (62) of MS patients. However, more recent studies showed the presence of JCV-DNA in both CSF (63, 64) and blood (65) in MS patients (although in a low percentage) and not in controls.

Moreover, novel clinical entities without typical PML lesions caused by JCV variants infecting cerebellar granule cell neurons and cortical pyramidal neurons as well as JCV meningitis have been recently discovered (66).

Therefore, JCV might be a feasible etiological candidate for both MS and brain tumors due to its ability to persist in the latent state mainly in myelin-producing oligodendrocytes and to its oncogenic capacity. However, JCV presence might still simply reflect the subclinical immunodeficiency of cancer patients or MS patients treated with immunosuppressive drugs.

## Therapy of Associated MS and Brain Tumors

There are no trials on the treatment of associated cases of MS and brain tumors owing to their rarity. The data here reported represent case reports and experimental observations.

It should not seem strange that the immunosuppressive treatments given for neoplasms may reduce disease activity in MS. In a patient of ours with MS and oligodendroglioma, MS activity significantly reduced both clinically and at MRI during the 2-year treatment with temozolomide (personal unpublished data).

Although the safety of radiotherapy for MS course was described in one case report (67), the negative effects of surgery and radiotherapy on MS, due to the liberation of brain-specific antigens triggering disimmune reactions, have also been reported (68). A vast review on MS relapses considered cranial radiation as their promoting factor (69).

Furthermore, a sphingosine analog FTY720, which downregulates the expression of sphingosine-1-phosphate receptors, is not only effective in MS (70) but also causes *in vitro* apoptosis of brain tumor stem cells derived from human glioblastoma tissue (71). Also dimethyl-fumarate, demonstrated to be effective in treatment of MS (72), appears to act on malignant brain neoplasms *in vitro* by reducing the proliferation rate, generating cell lysis, decreasing the expression of NF-κB, and restricting the growth of CD133 cells in gliomas (73).

## Conclusion

Although the co-existence of MS and brain tumors has been long described, many doubts regarding their possible causal association persist. JCV is of significant interest in this issue due to its ability to latent persist in the CNS and to its experimental neurooncogenic potential. Since MS is caused by putative CNS autoimmune mechanisms whereas brain neoplasms may be dependent on a subclinical immunosuppressive state, these pathologies can coexist only in particular situations. It can be hypothesized that these conditions may occur during the remyelinating processes coinciding with a decline of the CNS immune reaction and with the production of growth factors in the effort to repair the damage, thus favoring a hypothetical JCV-related cell neoplastic transformation in genetically or environmentally induced susceptible individuals. Studying with special care, the patients affected by both diseases, which apparently locate at the opposite ends of immunosurveillance, could allow to find the key to their pathogenesis.

## Conflict of Interest Statement

The authors declare that the research was conducted in the absence of any commercial or financial relationships that could be construed as a potential conflict of interest.
